# Predictors of Imminent Risk of Nonvertebral Fracture in Older, High‐Risk Women: The Framingham Osteoporosis Study

**DOI:** 10.1002/jbm4.10129

**Published:** 2019-01-18

**Authors:** Marian T Hannan, Derek Weycker, Robert R McLean, Shivani Sahni, Rebecca Bornheimer, Richard Barron, Thomas G Travison, Douglas P Kiel

**Affiliations:** ^1^ Institute for Aging Research Hebrew SeniorLife Department of Medicine Beth Israel Deaconess Medical Center, and Harvard Medical School Boston MA USA; ^2^ Policy Analysis Inc. (PAI) Brookline MA USA; ^3^ Corrona, LLC, Waltham, MA, USA, and Institute for Aging Research Hebrew SeniorLife Boston MA USA; ^4^ Amgen Thousand Oaks CA USA

**Keywords:** NON‐VERTEBRAL FRACTURE, OLDER ADULTS, IMMINENT FRACTURE, RISK PREDICTION, COHORT STUDY

## Abstract

Osteoporosis treatment decisions are often based solely on BMD or on 10‐year fracture risk; little is known about factors increasing imminent fracture risk. Understanding factors contributing to imminent risk of fracture is potentially useful for personalizing therapy, especially among those at high risk. Our aim was to identify predictors of nonvertebral fracture for 1‐ and 2‐year periods in women at high risk for fracture. The Framingham Osteoporosis Study cohort included 1470 women (contributing 2778 observations), aged ≥65 years with BMD hip *T*‐score ≤ −1.0, or history of fragility fracture (irrespective of *T*‐score). Nonvertebral fractures were ascertained prospectively over 1 year and 2 years following a baseline BMD scan. Potential risk factors included age, anthropometric variables, comorbidities/medical history, cognitive function, medications, history of fracture, self‐rated health, falls in the past year, smoking, physical performance, hip BMD *T*‐score, Activities of Daily Living (ADL) score, and caffeine and alcohol intakes. Predictive factors with *p* value ≤ 0.10 in bivariate Cox proportional hazards regression models were subsequently considered in multivariable models. Mean baseline age was 75 years (SD 6.0). During 1‐year follow‐up, 89 nonvertebral fractures occurred; during 2‐year follow‐up, 176 fractures occurred. Of the variables considered in the bivariate models, significant predictors of nonvertebral fractures included age, history of fracture, self‐rated health, falls in the prior year, BMD *T*‐score, ADL, renal disease, dementia, and current use of nitrates, beta‐blockers, calcium channel blockers, or antidepressants. In multivariable models, significant independent risk factors were history of fracture, self‐rated health, hip BMD *T*‐score, and use of nitrates. Significant 1‐year results were attenuated at the 2‐year follow‐up. In addition to the traditional factors of BMD and fracture history, self‐rated health and use of nitrates were independently associated with imminent risk of fracture in older, high‐risk women. These specific risk factors thus may be useful in identifying which women to target for therapy. ©2018 The Authors *JBMR Plus* published by Wiley Periodicals, Inc. on behalf of American Society for Bone and Mineral Research.

## Introduction

Rates of fractures of the hip, forearm, vertebrae, humerus, pelvis, and ankle increase with advancing age, especially after age 75 years.^1^ Lifetime risk of symptomatic fracture for a 50‐year‐old white woman has been estimated to be 13% for the forearm and 14% for the hip, several‐fold higher than corresponding risks among men of similar age.^2^, ^3^ These fractures are a major cause of morbidity, mortality, and healthcare costs, especially those involving the hip. Excess mortality is often seen in the year following a fracture in older adults.^3^ Loss of cortical and trabecular bone mass with advancing age, and resulting osteoporosis and associated bone fragility, is widely considered to be the major cause of fractures in older people.

In light of the substantial clinical and economic burden of osteoporotic fractures, the National Osteoporosis Foundation (NOF) promulgated guidelines for fracture prevention in 2010.^4^ In addition to nonpharmacological recommendations and maintenance of adequate intake of calcium and vitamin D, specific pharmacologic therapy (eg, bisphosphonates, calcitonin) is recommended for persons with: (1) BMD *T*‐scores ≤‐2.5; and (2) *T*‐scores between −1.0 and −2.5, and 10‐year predicted risk of hip fracture ≥3% or major osteoporosis‐related fracture ≥20% based on WHO's absolute Fracture Risk Assessment Tool (FRAX). The FRAX model is based on 12 risk factors, one of which is hip BMD, but a risk score can be calculated with or without information on BMD.

Despite endorsements of FRAX by both NOF and WHO, it has a number of limitations: (1) the general nature of some of the items (eg, fracture history does not take into account the timing or number of fractures); (2) the exclusion of falls information^5^, ^6^; (3) a relatively low area‐under‐the‐curve (AUC) in validation studies (only about 60% for major osteoporosis‐related fracture); and (4) application only to persons who are untreated for osteoporosis.^7^, ^8^ The importance of some of the items in FRAX also may be questionable. One study, for example, reported that there was no significant difference in predictive accuracy as measured by AUC between FRAX and simple models based only on BMD and age.^9^ Finally, 10‐year fracture risk is not necessarily indicative of short‐term fracture risk because the annual incidence of fracture increases substantively with age; thus, the risk in the first year of a given 10‐year interval is undoubtedly lower than that in year 10. Moreover, the relative risk of fracture for those with (versus without) a history of fracture is highest in the period soon after the event (ie, within the 1‐ to 2‐year period) and declines thereafter.^10^ Identifying these patients can reinvigorate the treatment discussions in this undertreated population.

For older women with established osteoporosis, short‐term risk prediction may be much more important than 10‐year risk, especially within the context of decisions regarding the use of new, high‐cost bone anabolic agents. Moreover, the importance of age, BMD, and other risk factors may be different in the prediction of short‐term fracture risk among older women with established osteoporosis compared with the prediction of long‐term fracture risk among the general population of postmenopausal women. Identification of women with high risk of fracture may be especially important in elderly women with osteoporosis or osteopenia. To better understand the risk factors for imminent (ie, within 1 to 2 years) fracture in this population, we conducted a cohort study using prospectively collected data from the large, well‐characterized Framingham Osteoporosis Study cohort. Our objective was to determine the factors that are independently associated with imminent nonvertebral fracture, for 1‐year and 2‐year time frames, in women aged 65 years and older, and at high risk of fracture.

## Participants and Methods

We used a cohort study design using previously collected prospective data to discern predictive factors associated with subsequent nonvertebral fractures in older women from a large, well‐characterized cohort, the Framingham Osteoporosis Study. The institutional review board at Hebrew SeniorLife approved this study; all participants signed an informed consent form prior to study enrollment.

### Participants

**Figure 1:**
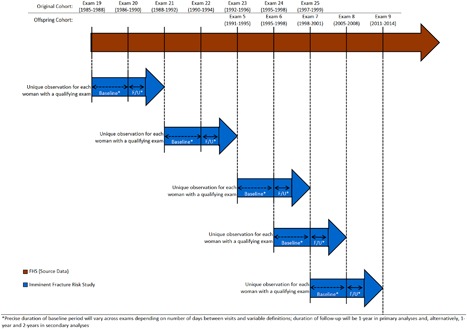
Schema of study design showing possible multiple observations per study participant.

The Framingham Study Original Cohort is a large, longitudinal population‐based study that in 1948 enrolled two‐thirds of the adults in the town of Framingham, Massachusetts, USA. In 1971 the Framingham Offspring Study enrolled the adult offspring (and their spouses) of the original cohort participants. Framingham Study participants undergo an extensive clinical examination and questionnaire battery every 2 or 4 years, depending upon the cohort. DXA scans of the hip and spine were obtained up to 3 times every 2 years between 1986 and1998 for the original cohort, and up to 3 times every 4 years between 1996 and 2008 for the offspring cohort, using a Lunar DPX‐L densitometer (GE Lunar Corp, Madison, WI, USA). For the current study, each BMD assessment was considered as the unit of observation, with the date of BMD assessment as baseline for the 1‐ and 2‐year fracture follow‐up periods. Thus, the same individual could have up to three separate observations included in the study sample for analysis (see Fig. 1). Our sample included women from both cohorts who were aged 65 years and older, and met at least one of the following criteria at baseline: (1) having osteoporosis defined as a *T*‐score ≤ −2.5 at the femoral neck or lumbar spine; (2) osteopenia defined as a *T*‐score > −2.5 to ≤ −1.0 at the femoral neck or lumbar spine; or (3) a history of nonvertebral or vertebral fracture, regardless of *T*‐score. These selection criteria are based in large part on the NOF treatment guidelines.^4^, ^11^ The study source sample comprised 1010 original cohort participants and 3025 offspring cohort participants. Of these women, 1470 met the study eligibility criteria.

### Assessment of fractures

Fracture of any nonvertebral site was defined as first occurrence during the follow‐up period of fracture of the hip, leg, knee, ankle, foot, clavicle, humerus, elbow, hand (excluding fingers), pelvis, or rib. Follow‐up information on mortality and fracture is complete for well over 95% of the cohort. Fracture ascertainment is performed through review of all participant contacts or study visits during which all hospitalizations and physician contacts are reported, including any fractures that occurred since their last visit. Fractures are adjudicated through an ongoing process using several overlapping sources including the clinic exam, fracture logs, hospitalization and death records, discharge summaries, reports from emergency department visits, operative reports, radiographic procedures, medical history updates, and other medical reports. The circumstances of each fracture are documented (degree of trauma, details of event, and treatment). Pathological fractures and fractures caused by high‐energy trauma (motor vehicle accident or assault) were excluded because it is not hypothesized that these high‐impact fractures will be related to factors for fragility fractures.

### Covariables

Each participant contributed up to three separate risk factor assessments, each considered in light of 1‐ and 2‐year risks of subsequent fracture. Potential predictive factors for fracture were evaluated at each eligible BMD assessment and included age, BMD, BMI, history of fracture, falls in the past 12 months, alcohol use, smoking status, caffeine use, history of medical comorbidities (eg, cardiovascular disease [CVD]), medication use (eg, anticonvulsants, benzodiazepines, bisphosphonates, and other osteoporosis drugs), cognitive function as assessed by the Mini‐Mental Status Examination (MMSE) score,^12^ physical function as assessed by self‐reported ability to perform activities of daily living (ADLs), and instrumental activities of daily living (IADLs), observed physical performance assessed by chair stands and measured walks, self‐rated health^13^, ^14^, ^15^, ^16^ (queried as excellent, good, fair, or poor), and depressive symptom (Center for Epidemiologic Studies Depression Scale [CES‐D]) score. Categorizing persons scoring ≥16 on the CES‐D as exhibiting depression symptoms has been validated in a general population, as well as in elderly persons.^17^, ^18^, ^19^, ^20^, ^21^ Candidate risk factors were based largely on those identified in a review of published literature on predictors of fractures and/or falls, and an ongoing similar evaluations presented in abstract form at scientific meetings.^3^, ^22^, ^23^, ^24^, ^25^

### Follow‐up

Following each risk factor assessment, 1‐ and 2‐year follow‐up periods were assessed for fracture occurrence. The one‐year follow‐up period began on the day after the date of the risk factor assessment and DXA scan, and ended at the date of occurrence of fracture, last contact, death, or 365 days later, whichever occurred earliest. For the 2‐year follow‐up, the maximum duration of follow‐up was 730 days. Only one (ie, the first) fracture event in each follow‐up interval for an observation was considered; multiple first fracture events occurring in different follow‐up periods for the same participant were considered (see Fig. 1).

### Statistical analysis

Each qualifying examination for each woman in the study population was considered as a separate observation in analyses, assessed over 1‐ and 2‐year follow‐up periods. Each participant contributed up to three such groupings of baseline and follow‐up observations. All observations were considered in a single analysis, taking into account clustering at the level of the participant based on repeated assessments. Risk factors were allowed to vary by assessment, such that a participant's age, comorbidities, etc., at each assessment were taken into account.

Potential risk factors that were continuous in nature were also evaluated using multilevel categorical (ie, ordinal or interval) variables; thresholds separating categories for a given factor were defined based on the quantiles of the distribution, and subsequently modified based on clinical and/or statistical considerations. Crude risk (numbers of participants with fracture events divided by person‐years or risk) of fracture were computed, and estimated relative risks were obtained comparing each variable grouping to referent categories. Following this, we modeled the risk of fracture using a clustered Cox proportional hazards model with robust variance estimated, employed to obtain validity in the presence of repeated assessments and outcomes.^26^ All risk factors with a *p* value <0.10 in the bivariate analyses were initially included simultaneously in a single multivariable model; grouped multilevel factors were included if any level had a *p* value <0.10. Variables that were no longer significant or important predictors in the multivariate context were excluded from the model (ie, other than age, no variables were retained based on a priori considerations), and the final parsimonious model was calculated. The importance of interactions between potential risk factors was examined. Model discrimination was evaluated based on the concordance index (c‐index).

## Results

There were 1470 Framingham Study women who met the study criteria of age ≥ 65 years with a baseline DXA indicating either osteoporosis (*T*‐score ≤−2.5), osteopenia (*T*‐scores for osteopenia were >−2.5 and included values as high as −1.0), or having a history of nonvertebral or vertebral fracture, regardless of *T*‐score. Mean age was 75 years (±5‐year SD). Table 1 shows the baseline characteristics of the study sample. These 1470 women contributed 2778 observations (up to three per woman) to both the 1‐ and 2‐year follow‐up analyses. Of these observations, 612 women contributed one observation, 408 women contributed two observations, and 450 women contributed three observations.

**Table 1:** Characteristics of the First Observation for Women in the Study Sample, Aged ≥65 Years With Osteoporosis, Osteopenia, or Fracture History (*N* = 1470)

Characteristic	
Age, mean ± SD, (years)	75.4 ± 6.0
Age category, *n* (%)	
<75 years	674 (45.9)
75–84 years	686 (46.7)
85+ years	110 (7.5)
Weight, mean ± SD (pounds)	144.2 ± 28.5
Height, mean ± SD (inches)	61.7 ± 2.5
BMI, mean ± SD (kg/m^2^)	26.7 ± 5.0
Hip BMD *T*‐score, mean ± SD	−2.20 ± 0.83

During the 1‐year follow‐up, 89 nonvertebral fractures occurred; during the 2‐year follow‐up, 176 fractures occurred. Hip fracture as well as wrist/forearm was the most frequent type of fracture in the 1‐year follow‐up (Table 2). Of 31 variables considered in unadjusted, bivariate models, those associated (*p* < 0.10) with nonvertebral fracture included age, history of fracture, self‐rated health, falls in the prior year, BMD *T*‐score, ADL score, renal disease, dementia, and current use of nitrates, beta‐blockers, calcium channel blockers, or antidepressants (Tables 3, 3b). There were no statistically significant interactions.

**Table 2:** Types of Nonvertebral Fractures Experienced Over Follow‐Up in Study Women

	Frequency at 1 year	Frequency at 2 year
Hi	18	33
Wrist/forearm	18	33
Foot/ankle/leg	15	34
Upper arm/shoulder	16	28
Ribs	10	14
Other	12	34
Total	89	176

**Table 3:** One‐Year Follow‐Up Bivariate Analyses of Potential Risk Factors and Nonvertebral Fracture in Women Aged ≥65 Years With Osteoporosis, Osteopenia, or Fracture History

		Women with osteoporosis, osteopenia, fracture history (*n* observations = 2778)						
						Cox PH model		
Covariates		No. of observations	No. of Fx	% Fx	Rel. risk	HR	95% CI	*p* value
Age								
<75 years		1274	32	2.5	REF			
75–84 years		1296	47	3.6	1.5	1.5	0.93, 2.28	0.10
85+ years		208	10	4.8	1.9	1.9	0.95, 3.92	0.07
History of fracture								
Yes		939	41	4.4	1.7	1.7	1.12, 2.57	0.01
No		1839	48	2.6	REF	REF		
Falls in last year								
0		1585	41	2.6	REF	REF		
1		467	22	4.7	1.8	1.8	1.09, 3.07	0.02
2+		636	24	3.8	1.5	1.5	0.88, 2.41	0.14
Smoking								
No, not current		2539	81	3.2	REF	REF		
Yes, current		237	8	3.4	1.1	1.1	0.51, 2.17	0.90
Estrogen use								
No		2391	82	3.4	REF	REF		
Yes, current		235	4	1.7	0.5	0.5	0.18, 1.33	0.16
Yes, former		150	3	2.0	0.6	0.6	0.19, 1.88	0.37
Total ADL score								
0–2 Severe impairment		6	1	16.7	5.4	6.2	0.86, 44.6	0.07
3–4 Moderate impairment		18	1	5.6	1.8	1.8	0.25, 13.1	0.55
5‐6 Full function		2730	85	3.1	REF	REF		
Self‐related health								
Excellent		886	17	1.9	REF	REF		
Good		1486	53	3.6	1.9	1.9	1.08, 3.21	0.03
Fair		325	13	4.0	2.1	2.1	1.02, 4.33	0.04
Poor		26	3	11.5	9.7	6.0	1.8, 20.4	0.004
*T*‐score category								
≤ −2.5 or lower (osteoporosis)		980	54	5.5	3.3	3.3	0.80, 13.5	0.10
> −1.0 to −2.499 (osteopenic)		1680	33	2.0	1.2	1.2	0.3, 4.81	0.84
≥ −1.0 or better (normal)		118	2	1.7	REF	REF		
Medical history/comorbidities								
Hypertension	Yes	1420	40	2.8	0.8	0.8	0.50, 1.15	0.19
	No	1318	48	3.6	REF	REF		
Renal disease	Yes	169	9	5.3	1.8	1.8	0.92, 3.66	0.08
	No	2597	78	3.0	REF	REF		
Emphysema	Yes	70	4	5.7	1.8	1.8	0.67, 4.99	0.24
	No	2698	85	3.2	REF	REF		
Degenerative joint disease	Yes	1032	32	3.1	1.0	1.0	0.63, 1.50	0.90
	No	1722	55	2.9	REF	REF		
Dementia	Yes	50	3	6.0	1.9	2.0	0.62, 6.19	0.25
	No	2689	84	3.1	REF	REF		
High thyroid	Yes	188	6	3.2	0.9	0.9	0.41, 2.18	0.90
	No	1965	68	3.5	REF	REF		
Parkinson disease	Yes	10	0	0.0	‐‐	‐‐		
	No	2103	72	3.4				
Diabetes	Yes	214	8	3.7	1.2	1.2	0.59, 2.50	0.61
	No	2564	81	3.2	REF	REF		
CVD	Yes	1261	48	3.8	1.4	1.4	0.93, 2.13	0.11
	No	1517	41	2.7	REF	REF		
Cancer	Yes	651	18	2.8	0.8	0.8	0.50, 1.40	0.49
	No	2127	71	3.3	REF	REF		
Medication use								
Nitroglycerine	Yes	90	3	3.3	0.9	1.0	0.31, 3.14	0.99
	No	2416	82	3.4	REF	REF		
Nitrates	Yes	98	11	11.2	3.7	3.9	2.06, 7.31	<.0001
	No	2408	74	3.1	REF	REF		
Beta blockers	Yes	471	23	4.9	1.6	1.6	1.01, 2.63	0.05
	No	2040	62	3.4	REF	REF		
Calcium channel blocker	Yes	385	19	4.9	1.6	1.6	0.96, 2.67	0.07
	No	2126	66	3.1	REF	REF		
Diuretics	Yes	516	14	2.7	0.8	0.8	0.44, 1.39	0.40
	No	1946	67	3.4	REF	REF		
Anticholesterol	Yes	412	9	2.2	0.6	0.7	0.33, 1.30	0.22
	No	2362	80	3.4	REF	REF		
Thyroid med	Yes	401	9	2.2	0.6	0.6	0.31, 1.24	0.18
	No	2104	76	3.6	REF	REF		
Oral glucocorticoids	Yes	58	2	3.4	0.9	0.9	0.23, 3.75	0.91
	No	1920	71	3.7	REF	REF		
Anti‐anxiety	Yes	121	5	4.1	1.2	1.2	0.50, 3.03	0.66
	No	2393	80	3.3	REF	REF		
Sleeping	Yes	45	2	4.4	1.3	1.4	0.33, 5.52	0.67
	No	2469	83	3.7	REF	REF		
Antidepressants	Yes	132	8	6.1	1.9	1.9	0.91, 3.92	0.09
	No	2381	77	3.2	REF	REF		
Anticonvulsants	Yes	21	1	4.8	1.3	1.2	0.17, 8.97	0.83
	No	1852	69	4.2	REF	REF		
Progesterone	Yes	83	1	1.2	0.4	0.4	0.05, 2.61	0.31
	No	2677	88	3.3	REF	REF		

Any sum <2778 observations is based on missing values of a variable.

FX = fractures; ADL = activities of daily living; CVD = cardiovascular disease; REF = Referent Category.

**Table 4:** Two‐Year Follow‐Up Bivariate Analyses of Potential Risk Factors and Nonvertebral Fracture (2 Year) in Women Aged ≥65 Years With Osteoporosis, Osteopenia, or Fracture History

		Women with osteoporosis, osteopenia, fracture history (*n* = 2778)						
						Cox PH model		
Covariates		No. of observations	No. of Fx	% Fx	Rel. risk	HR	95% CI	*p* value
Age								
<75 years		1274	63	4.9	REF			
75–84 years		1296	97	7.5	1.5	1.5	1.12, 2.12	0.007
85+ years		208	16	7.7	1.6	1.6	0.93, 2.80	0.09
History of fracture								
Yes		939	75	8.0	1.5	1.5	1.11, 2.01	0.009
No		1839	101	5.5	REF	REF		
Falls in last year								
0		1585	96	6.1	REF	REF		
1		467	39	8.4	1.4	1.4	0.96, 2.03	0.08
2+		636	36	5.7	0.9	0.9	0.63, 1.35	0.68
Smoking								
No, not current		2539	161	6.3	REF	REF		
Yes, current		237	15	6.3	1.0	1.0	0.58, 1.68	0.96
Estrogen use								
No		2391	161	6.7	REF	REF		
Yes, current		235	8	3.4	0.5	0.5	0.24, 0.99	0.05
Yes, former		150	7	4.7	0.7	0.7	0.34, 1.53	0.39
Total ADL score								
0–2 Severe impairment		24	4	16.7	2.4	2.6	0.97, 7.10	0.06
3–4 Moderate impairment		620	20	3.2	0.5	0.5	0.31, 0.78	0.003
5–6 Full function		2110	149	7.1	REF	REF		
Self‐related health								
Excellent		886	42	4.7	REF	REF		
Good		1486	96	6.5	1.4	1.4	0.95, 1.95	0.10
Fair		325	29	8.9	1.9	1.9	1.21, 3.12	0.006
Poor		26	3	11.5	2.4	2.5	0.78, 8.08	0.12
*T*‐score category								
≤ −2.5 or lower (osteoporosis)		980	94	9.6	2.3	0.4	0.18, 1.08	0.07
> −1.0 to −2.499 (osteopenic)		1680	77	4.6	1.1	0.5	0.35, 0.63	<0.0001
≥ −1.0 or better (normal)		118	5	4.2	REF	REF		
Medical history/comorbidities								
Hypertension	Yes	1427	85	6.0	0.9	0.9	0.65, 1.19	0.40
	No	1318	88	6.7	REF	REF		
Renal disease	Yes	169	14	8.3	1.3	1.4	0.82, 2.45	0.21
	No	2597	160	6.2	REF	REF		
Emphysema	Yes	70	7	10.0	1.6	1.6	0.77, 3.49	0.20
	No	2698	169	6.3	REF	REF		
Degenerative joint disease	Yes	1032	65	6.3	1.0	1.0	0.73, 1.36	0.99
	No	1722	109	6.3	REF	REF		
Dementia	Yes	50	6	12.0	1.9	2.0	0.88, 4.48	0.10
	No	2689	167	6.2	REF	REF		
High thyroid	Yes	188	12	6.4	1.0	1.1	0.58, 1.91	0.86
	No	1965	125	6.4	REF	REF		
Parkinson	Yes	10	0	0.0	‐‐	‐‐		
	No	2103	134	6.4				
Diabetes	Yes	214	14	6.5	1.0	1.1	0.63, 1.88	0.76
	No	2564	162	6.3	REF	REF		
CVD	Yes	1261	88	7.0	1.2	1.2	0.89, 1.60	0.24
	No	1517	88	5.8	REF	REF		
Cancer	Yes	651	39	6.0	0.9	0.9	0.66, 1.35	0.76
	No	2127	137	6.4	REF	REF		
Medication use								
Nitroglycerine	Yes	90	7	7.8	1.2	1.2	0.56, 2.55	0.64
	No	2416	160	6.6	REF	REF		
Nitrates	Yes	98	14	14.3	2.3	2.5	1.43, 4.27	0.001
	No	2408	153	6.4	REF	REF		
Beta blockers	Yes	471	40	8.5	1.4	1.4	0.98, 1.99	0.07
	No	2040	127	6.2	REF	REF		
Calcium channel blockers	Yes	385	33	8.6	1.4	1.4	0.95, 2.04	0.09
	No	2126	134	6.3	REF	REF		
Diuretics	Yes	516	36	7.0	1.1	1.1	0.74, 1.56	0.69
	No	1946	1257	6.4	REF	REF		
Anticholesterol	Yes	412	21	5.1	0.8	0.8	0.51, 1.27	0.36
	No	2362	155	6.6	REF	REF		
Thyroid med	Yes	401	27	6.7	1.0	1.0	0.68, 1.54	0.92
	No	2104	139	6.6	REF	REF		
Oral glucocorticoids	Yes	58	6	10.3	1.5	1.5	0.67, 3.45	0.32
	No	1920	131	6.8	REF	REF		
Anti‐anxiety	Yes	121	6	5.0	0.7	0.7	0.33, 1.66	0.46
	No	2393	161	6.7	REF	REF		
Sleeping	Yes	45	5	11.1	1.7	1.8	0.72, 4.26	0.22
	No	2469	162	6.6	REF	REF		
Antidepressants	Yes	132	12	9.1	1.4	1.4	0.78, 2.53	0.26
	No	2381	155	6.5	REF	REF		
Anticonvulsants	Yes	21	3	14.3	2.1	2.1	0.67, 6.65	0.20
	No	1852	124	6.7	REF	REF		
Progesterone	Yes	83	3	3.6	0.6	0.6	0.18, 1.71	0.30
	No	2677	172	6.4	REF	REF		

Any sum <2778 observations is based on missing values of a variable.

FX = fractures; ADL = activities of daily living; CVD = cardiovascular disease; REF = Referent Category.

In the 1‐year multivariable model, significant independent risk factors were history of fracture, self‐rated health, hip BMD *T*‐score, and use of nitrates (Table 5). Significant associations from the 1‐year time frame were mostly attenuated when considered over 2 years of follow‐up. Discrimination of the 1‐year model, based on the c‐index, was 0.71 (SE 0.03), indicating good discrimination, with the 2‐year model c‐index of 0.64 (SE 0.02).

**Table 5:** Multivariable Analysis of Potential Risk Factors and Risk of Nonvertebral Fracture Over 1 Year and 2 Years of Follow‐Up (*n* Observations = 2778)

	1‐year fracture risk Cox PH model					2‐year fracture risk Cox PH model				
	*N*	Fx	HR	95% CI	*p* value	*N*	Fx	HR	95% CI	*p* value
Age										
<75 years	1274	32	REF			1274	63	REF		
75–84 years	1296	47	1.0	0.58,1.57	0.85	1296	97	1.2	0.85,1.73	0.29
85+ years	208	10	1.1	0.49,2.26	0.90	208	16	1.0	0.57,1.92	0.89
History of fracture										
Yes	939	41	1.4	0.89,2.19	0.14	939	75	1.4	1.00,1.91	0.05
No	1839	48	REF			1839	101	REF		
ADL score										
0–4 impaired	24	2	0.9	0.12,6.57	0.91	24	4	0.6	0.08,4.40	0.62
5–6 functional	2730	85	REF			2730	169	REF		
Self‐rated health										
Excellent	886	17	REF			886	42	REF		
Good/fair	1811	66	1.5	0.84,2.75	0.16	1811	125	1.4	0.91,2.00	0.13
Poor	26	3	4.0	1.10,14.3	0.04	26	3	2.1	0.64,7.05	0.22
BMD *T*‐score										
≤ −2.5 (osteoporosis)	980	54	2.8	1.75,4.54	<.0001	980	94	2.0	1.46,2.81	<.0001
> −1.0 (osteopenic/normal)	1798	35	REF			1798	82	REF		
Falls in past year										
Yes 1+	1103	46	1.3	0.83,2.04	0.25	1103	75	1.0	0.66,1.27	0.60
No	1585	41	REF			1585	96	REF		
Medication use in last year										
Nitrates										
Yes	98	11	2.6	1.22,5.39	0.01	98	14	1.8	0.95,3.40	0.07
No	2408	74	REF			2408	153	REF		
Beta blockers										
Yes	471	23	1.3	0.78,2.20	0.30	471	40	1.2	0.82,1.76	0.36
No	2040	62	REF			2040	127	REF		
Calcium channel blockers										
Yes	385	19	1.1	0.60,1.92	0.82	385	33	1.1	0.75,1.74	0.55
No	2126	66	REF			2126	134	REF		
Antidepressants										
Yes	132	8	1.7	0.76,3.64	0.20	132	12	1.3	0.72,2.47	0.36
No	2381	77	REF			2381	155	REF		

Concordance index (SE) for 1‐year model is 0.71 (0.03) and for 2‐year model is 0.64 (0.02). Each variable adjusted in models for all other listed variables.

FX = fractures; REF = Referent Category.

## Discussion

The purpose of this study was to better understand the risk factors for imminent nonvertebral fracture in a population of older postmenopausal women with osteoporosis, osteopenia, and/or fracture history in whom fracture risk may be elevated. This study found several factors were independently associated with the 1‐year risk of fracture including history of fracture, poor self‐reported health status, osteoporosis indicated by *T*‐score level, and use of nitrates in the past 2 years or more. Additional factors including falls history, renal disease, and use of antidepressants met our bivariate criterion for inclusion in the multivariate model, but were not statistically significant once other variables were considered. Several recent studies have also examined risk of imminent fracture and report similar factors derived from claims databases and clinical studies. Two recent studies^10^, ^24^ on the 1‐ and 2‐year risk from the Medicare 20% sample and the Truven Commercial and Medicare Claims Dataset supported the importance of older age, history of other adult fracture, prior recent falls, poorer health status, diagnosis of osteoporosis, and comorbidities that trigger more frequent falls (Alzheimer disease, CNS diseases), as well as medications and equipment that linked to poorer cognition, physical function, and motor skills (use of wheelchair, walker, cane, narcotics, centrally sedating anticholinergic medications, and sedative hypnotic medications). Other research from observational cohorts (Study of Fractures, Canadian Multi‐Center Osteoporosis Study, Kaiser, Swedish Registry Data) have demonstrated similar findings with older age, BMD *T*‐score, prior fracture, falls, and falls‐related risk factors (comorbidities, medications) being the dominant predictors. The results support many of the fracture risk prediction tools that focus on longer term risk prediction (5 to 10 years) with the exception that falls and fall‐related factors (diseases and medications) are also quite important. Currently, only tools such as Q‐Fracture capture these important risk factors, whereas FRAX and others do not consider them. Previous research has reported that falls represent at least 30% of the risk of fracture, which would be accurate for these risk factors to factor so consistently and prominently into defining the imminent risk fracture patient across data source and type.^5^ Imminent (1 to 2 year) risk of fracture appears to be an important, yet relatively understudied, time frame that may be relevant to stimulate more patient interest in therapeutics aimed at fracture prevention.

There is a strong inverse relationship between bone density and risk of fracture, with a two‐ to threefold increased risk per standard deviation decline in BMD. Nonetheless, at any given level of BMD, fracture risk increases with advancing age, highlighting the fact that factors other than bone density are independently related to risk of fracture. Although some of these factors affect skeletal integrity (eg, bone turnover, trabecular architecture), nonskeletal factors may also play an important role to the extent that they increase the risk of falls, which are the precipitating factor in the vast majority of osteoporotic fractures.

Not surprisingly, falls were predictive of imminent fracture risk in our study despite data on falls occurrence being somewhat limited in scope (self‐reported yes/no for past year at BMD scan visit). Once other important variables were considered in our study, however, falls were no longer statistically significant in the multivariable model. A 2016 case‐control study of short‐term fracture risk using US claims data reported higher imminent fracture risk for older adults with falls, poor health, specific comorbidities, psychoactive medication use, and mobility impairment.^10^ A 2017 cohort study of women in the Study of Osteoporotic Fractures (SOF) also found prior falls as well as prior fracture, walking speed, Parkinson disease, smoking, and stroke to be predictive factors.^25^ A select list of medications used in the past year also predicted short‐term fracture risk in our study. The claims data study examined class of medications rather than specific medications, but reported similar findings for antidepressants^10^; however, our study did not have sufficient numbers of users of medication categories to closely examine many medications. Although a previous study found nitrates to protect against hip fracture,^27^ we found use in the past 2 years increased the risk of hip fracture. This could be because of the disease for which they were prescribed (CVD) or because of the hypotensive effects of taking nitrates. Because persons with CVD manifest by aortic calcification, they may also have greater fracture risk.^28^ The study from claims data supports the limited medications data from our cohort. Attributing risk to medication prescribing may suffer from bias by indication, making it quite difficult to discern the disease from the medication used to treat the disease, as the causal risk factor.

Understanding the factors that elevate short‐term fracture risk are important for identifying patients at imminent risk of fracture, as they merit prompt evaluation and treatment for osteoporosis. It is important to note that falls and other variables (as seen in Table 5) were most predictive of short‐term fracture risk in the 1‐year time frame than 2‐year follow‐up time.

Whether the risk factors of primary importance in a broad group of postmenopausal women (eg, older age and low BMD), retain their relative importance in this high‐risk subgroup is largely unknown. Perhaps for this group of women, identification of the subset at imminent risk of falling is much more important from the perspective of risk stratification. Moreover, in older postmenopausal women and in women with a recent fracture, ascertainment of risk factors for imminent fracture may have greater clinical relevance than identification of risk factors that have long‐term prognostic importance, but poorer predictive accuracy over the short‐run.

The NOF guidelines list more than 75 conditions, diseases, and medications that cause or contribute to osteoporosis and fractures; they also identify 21 risk factors for falls.^4^ Among the risk factors for falls that have been identified are muscle weakness, gait and balance deficits, visual impairment, arthritis, impaired ADLs, depression, cognitive impairment, and age >80 years.^29^ There have been numerous studies of these and other risk factors for osteoporotic fractures and falls. Major studies included those based on the SOF, the Framingham Osteoporosis Study, the National Osteoporosis Risk Assessment (NORA) Study, the Canadian Multicentre Osteoporosis Study (CaMos), Women's Health Initiative (WHI), the Million Women Study, and the Global Longitudinal Study of Osteoporosis in Women (GLOW). The current study results winnow this long list of potential factors to several that may be particularly pertinent for imminent risk of fracture.

The unadjusted findings of this study may indicate those items of interest to consider in a clinical examination or check‐up examination for older women at high‐risk of nonvertebral fracture. The parsimonious results from analyses considering all factors showed that history of fracture, poor self‐reported health status, osteoporosis indicated by *T*‐score level, and use of nitrates in the past 2 years or more were indicators of imminent nonvertebral fractures in our study. These factors may be of particular interest to clinicians and helpful in highlighting persons who may warrant strong consideration for falls‐prevention programs. Relatively sparse information (compared to long‐term fracture risk) exists in the published literature to compare with our results on imminent risk of nonvertebral fractures. The attenuation of results over 2 years compared to the results over 1 year suggests that the effects of risk factors diminish with time, even with this short time frame, such that the estimations of long‐term risk of fracture may not be as useful clinically in those at high risk.

### Strengths and limitations

A strength of this study is that the sample population was derived from the Framingham Study, a well‐characterized cohort that contains large numbers of women ages 65 years and older. Reproducibility of BMD measurement is good in studies such as the Framingham Study (coefficient of variations of 2% to 3% at the proximal femur); it may be worse in clinical practice where quality assurance factors and operator experience may be less. Thus, women in this study were unlikely to be misclassified in terms of their *T*‐score, and unlikely to be erroneously included or excluded from the study population. Another strength is the ability to focus on a large number of older women at high risk for fracture—the group most susceptible to long‐term risk of fracture as well. Also we were able to include consideration of many major factors that are likely to be available clinically, including comorbidities and medications.

However, a number of limitations need to be considered when interpreting the findings in this study. The Framingham Study is primarily comprised of whites; thus, the results may not be generalizable to non‐white groups. Although Framingham Study participants undergo examinations every 2 or 4 years and are followed for fractures, it is possible that not all fractures are captured and appropriately adjudicated. Another limitation is that several of the comorbidities included in our study had low prevalence (eg, only 50 subjects had medical history of dementia and only 6 of them had a subsequent fracture. Similarly, only 10 persons had Parkinson disease, limiting the statistical power to detect an imminent fracture risk for these conditions. Also, practice patterns, technology, and other largely unobservable or quantifiable factors may have changed over the period of observation in our cohort, which could have an impact upon measurements of risk factors over time and estimated relationships with fracture. Finally, information on risk factors was limited to those available at the Framingham Study examinations. Although use of selected medications is confirmed by interview and examination of medication containers, whether patients continued to take medications during follow‐up is uncertain. Although the timing of clinical fractures between visits is accurately detailed in the data source, subclinical or asymptomatic fractures may not be identified on a continuous basis in the Framingham Study cohorts. The true timing of such events thus is unknown. Also, this study did not examine risks for specific sites of fracture. In the Framingham Study cohorts, fractures are tracked via self‐report on an ongoing basis, and confirmed via medical records review. It thus is not possible to capture potentially important changes in time‐dependent risk factors that occurred between visits or prior to the occurrence of fracture during a given interval. The follow‐up interval used in our study was quite short, so this possible concern is unlikely to have had a major impact on our results. Yet, as such information is not available, the precise nature of the relationships between selected risk factors (eg, use of medications) and occurrence of fracture may be mischaracterized in analyses. Moreover, because not all self‐reported fractures are confirmed by medical records, some fractures were self‐reported only.

## Conclusion

In conclusion, this study found several factors were associated with the imminent 1‐year risk of nonvertebral fracture in older women at high risk for fracture. These results imply that the risk for fractures is increased in high‐risk women based on factors, in addition to BMD, that are easily obtained at a clinical visit. These factors may be useful in identifying which women to target for therapy and/or other interventions. Fractures remain devastating events for older adults. Insights from imminent fracture occurrences may lead to better interventions, especially for those women at high‐risk of future fracture. Future work should highlight the short‐term benefits of interventions on decreasing the risk for nonvertebral fractures.

## Disclosures

With the exceptions noted below, the authors have nothing to disclose. Drs Hannan and Kiel received research funding from Policy Analysis Inc. in the form of a research grant, but retained full independence in use and reporting of data generated from this funding. Dr Weycker and Ms Bornheimer are employed by Policy Analysis Inc. (PAI), which received funding for this research from Amgen. Mr Barron was an employee of Amgen and owns stock in Amgen. Dr Hannan has received a grant from Merck Sharp & Dohme for an unrelated investigator‐initiated project as well as support from Dairy Management Inc. for work unrelated to the current project. Dr Kiel has received a grant from Merck Sharp & Dohme for an unrelated investigator‐initiated project, support from Dairy Management Inc. for work unrelated to the current project, and has served on a scientific advisory board for Merck Sharp & Dohme. He has received royalties and stipends for publication by Wolters, Kluwer and Springer, Inc. Dr Sahni has received an institutional grant from Dairy Management Inc. for work unrelated to the current project, and serves on the National Dairy Council's Nutrition Research Scientific Advisory Committee.
